# Ethical implications of using artificial intelligence to support scientific writing

**DOI:** 10.5935/0004-2749.2025-0018

**Published:** 2025-02-11

**Authors:** Renato Ambrósio Jr., Alexandre Batista da Costa Neto, Matheus Puppe Magalhães, Milton Yogi, Kaio Pereira, Aydano Pamponet Machado

**Affiliations:** 1 Departamento de Oftalmologia, Universidade Federal do Estado do Rio de Janeiro, Rio de Janeiro, RJ, Brazil; 2 MPuppe & Associados Advogados, Brasília, DF, Brazil; 3 Benvista Oftalmologia, São Paulo, SP, Brazil; 4 Centro Universitário de Maringá, Maringá, PR, Brazil; 5 Computing Institute, Universidade Federal de Alagoas, Maceió, AL, Brazil

Dear Editor,

We commend the editorial “Challenges and Advantages of Being a Scientific Journal Editor in the Era of ChatGPT”^(1)^, for its timely insights on artificial intelligence (AI) in scientific publishing. We would like to further reflect on the lessons learned from past experiences and explore future possibilities for AI integration.

“*If it is not right, do not do it; if it is not true, do not say it*”- Marcus Aurelius, *Meditations*

This principle, as articulated by Marcus Aurelius, resonates with the ongoing Fourth Industrial Revolution, positioning AI as a tool to augment human judgment. However, it also emphasizes the need to safeguard the integrity of scientific writing.

The rapid advancement of technology presents several ethical challenges, particularly in the areas of authorship, transparency, and plagiarism. It is essential to disclose AI’s role in scientific research and writing. Researchers must use AI responsibly to enhance the language and structure of their work without compromising intellectual integrity. Transparency and vigilance are paramount, and tools such as those listed in table 1 can support high-quality writing. This letter itself is an example of the ethical potential of AI-enhanced communication.

**Table 1:** Current AI tools for enhancing scientific writing in medical imaging

Tool	Description	Features	Website
ChatGPT	Conversational AI by OpenAI	Natural language processing, text generation, interactive dialogue	openai.com/chatgpt
Perplexity.ai	AI-powered search engine	Answer generation, information retrieval, contextual understanding	perplexity.ai
Grammarly	AI-powered writing assistant	Grammar checking, style improvement, plagiarism detection	grammarly.com
DeepL Write	AI writing tool by DeepL	Advanced translation, writing assistance	deepl.com/write
Elicit	AI research assistant	Literature review assistance, question answering	elicit.org
QuillBot	AI paraphrasing and summarization tool	Paraphrasing, summarization, grammar checking	quillbot.com
Scholarcy	AI-powered article summarizer	Summarizes research papers, extracts key information	scholarcy.com
Writefull	AI tool for academic writing	Language feedback, automated paraphrasing, vocabulary suggestions	writefull.com
SciNote Manuscript Writer	AI tool for scientific writing	Assists in manuscript drafting and organization	scinote.net
LanguageTool	Multilingual grammar, style, and spell checker	Grammar checking, style improvement, multilingual support	languagetool.org

However, AI tools can perpetuate linguistic and stylistic biases, which could have far-reaching implications in scientific writing and healthcare outcomes. These biases arise from datasets, models, and other factors, requiring the implementation of effective strategies to mitigate them^(2)^. Ethical AI use must address these biases to ensure equitable and high-quality contributions to science. Additionally, there are legal concerns related to data protection, intellectual property, and compliance with regulations such as the General Data Protection Regulation (GDPR), which must be carefully managed. Both researchers and clinicians must adopt informed and ethical approaches when integrating AI into their work^(2)^.

AI is reshaping knowledge creation, creativity, and judgment in science and medicine. While it enhances efficiency and broadens access, there is a risk of standardizing thought and diminishing the value of expert knowledge. It is crucial to preserve diversity, critical analysis, and curiosity. Ethical frameworks must ensure that AI complements, rather than replaces, human input in scientific and medical discovery.

Furthermore, AI’s integration into scientific writing and medicine raises complex legal challenges, particularly in data protection, intellectual property rights, and adherence to regulations like GDPR. As the world becomes increasingly interconnected, the ethical and legal standards for AI use will evolve. Researchers and clinicians must remain agile in navigating issues such as authorship and plagiarism^(3)^. While AI is redefining norms, it cannot supplant human creativity and expertise. Legal frameworks are evolving in response to global communication and cultural shifts, highlighting the necessity for responsible and informed AI use^(3)^.

The ethical foundation of AI relies must be built on principles of beneficence, nonmaleficence, autonomy, justice, and explicability. These values promote well--being, fairness, transparency, and human oversight while preventing harm and addressing societal inequalities in writing and medicine. Clear ethical standards are critical to preserving integrity and safeguarding the rights of participants. Although AI’s role in enhancing creativity-like a research assistant-may prompt disclaimers regarding authorial integrity, its potential should not be undervalued^(4)^. A collaborative approach involving all stakeholders is essential for developing and updating ethical guidelines, ensuring that AI’s integration into scientific and medical domains does not undermine public trust.

**Figure 1:**
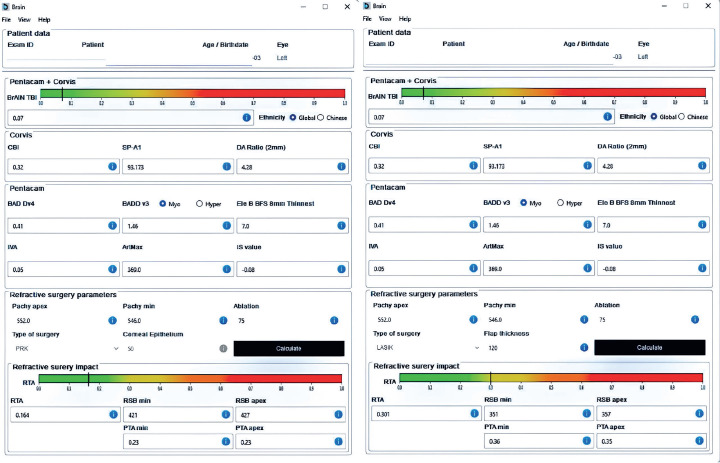
BrAIN-enhanced ectasia display of a 24-year-old refractive candidate considering surface ablation and LASIK planning. The display highlights moderate RTA despite the residual stromal bed being greater than 350 and the percentage of tissue altered being below 0.35. Based on these findings, custom photorefractive keratectomy (PRK) was deemed the more suitable option.

Beyond research and writing, AI tools such as the Brazilian Artificial Intelligence Networking (BrAIN)^(5)^ ectasia screening software demonstrates significant improvements in productivity and accuracy. This tool assists in clinical decision-making by integrating the cornea’s intrinsic susceptibility with the optimized Tomography and Biomechanics Index (TBI)^(6)^, along with the relational impact from laser vision correction procedures on relational tissue altered (RTA). However, it is important to emphasize that the software does not replace the physician’s responsibility but rather offers an objective, individualized risk assessment for ectasia progression, thereby enhancing the physician’s ability to make informed, patient-centered decisions (Figure 1).

Embracing AI responsibly requires a balanced approach that fosters innovation while maintaining ethical integrity. Addressing challenges such as bias, transparency, and legal compliance demands a proactive and reflective mindset. This letter proposes actionable frameworks, underscoring the need for robust guidelines, training, and interdisciplinary collaboration. AI should serve as a complement to human creativity and care, supported by ethical standards and education. We advocate for ongoing dialogue among editors, researchers, and policymakers to shape this evolving landscape while safeguarding the core values of humanity, curiosity, and integrity.
